# A Critical Review on the Complex Interplay between Social Determinants of Health and Maternal and Infant Mortality

**DOI:** 10.3390/children9030394

**Published:** 2022-03-10

**Authors:** Rada K. Dagher, Deborah E. Linares

**Affiliations:** National Institute on Minority Health and Health Disparities, National Institutes of Health, U.S. Department of Health and Human Services, Two Democracy Plaza, 6707 Democracy Blvd, Suite 800, Bethesda, MD 20817, USA; deborah.linares@nih.gov

**Keywords:** maternal mortality, infant mortality, health disparities, social determinants of health

## Abstract

Background: U.S. maternal and infant mortality rates constitute an important public health problem, because these rates surpass those in developed countries and are characterized by stark disparities for racial/ethnic minorities, rural residents, and individuals with less privileged socioeconomic status due to social determinants of health (SDoH). Methods: A critical review of the maternal and infant mortality literature was performed to determine multilevel SDoH factors leading to mortality disparities with a life course lens. Results: Black mothers and infants fared the worst in terms of mortality rates, likely due to the accumulation of SDoH experienced as a result of structural racism across the life course. Upstream SDoH are important contributors to disparities in maternal and infant mortality. More research is needed on the effectiveness of continuous quality improvement initiatives for the maternal–infant dyad, and expanding programs such as paid maternity leave, quality, stable and affordable housing, and social safety-nets (Medicaid, CHIP, WIC), in reducing maternal and infant mortality. Finally, it is important to address research gaps in individual, interpersonal, community, and societal factors, because they affect maternal and infant mortality and related disparities. Conclusion: Key SDoH at multiple levels affect maternal and infant health. These SDoH shape and perpetuate disparities across the lifespan and are implicated in maternal and infant mortality disparities.

## 1. Introduction

Maternal and infant mortality rates in the United States (U.S.) constitute an important public health problem, because these rates surpass those in similarly large, developed, and wealthy countries, and are characterized by stark disparities for people of color, underserved rural residents, and those of a low socioeconomic status. For example, although the global maternal mortality ratio (MMR) has decreased by 45% over the last three decades (385 per 100,000 live births in 1990 to 211 in 2017), the U.S. MMR (calculated as the number of maternal deaths per 100,000 live births) has increased by 58% (12 in 1990 to 19 in 2017) [1]. Every year, about 700 U.S. women die due to pregnancy and its complications [2]. Furthermore, there are significant disparities by race, where non-Hispanic Black women have a maternal mortality rate which is 3.55 times higher than that for non-Hispanic White women [3] and American Indian/Alaskan Native (AI/AN) women have a rate that is 2.3 times higher than that for non-Hispanic White women [2]. Socioeconomic disparities also exist, where women with fewer than 12 and 12 years of education have an MMR that is 2.1 and 2.5 times higher, respectively, than women with a college degree [4]. However, the educational gradient is mainly consistent for non-Hispanic White women, where those with fewer than 12 years of education experience 4.8 times higher maternal mortality than their college-educated counterparts [4]. Moreover, within each educational category, non-Hispanic Black women experience higher maternal mortality rates than non-Hispanic White women, with this racial disparity being most pronounced among college-educated women [4]. Regarding rural–urban disparities, the highest maternal mortality rates are found in small rural areas where the rates are 80% higher (relative ratio = 1.8) than those for women living in inner cities [4].

Severe maternal morbidity (SMM) has a higher prevalence than maternal mortality, affecting around 50,000 women per year (0.5 to 1.3% of pregnancies) [5]. SMM has been defined as “an unintended outcome of the process of labor and delivery that results in significant short- or long-term consequences to a woman’s health,” [6]. The U.S. Centers for Disease Control and Prevention define SMM as an index of 21 indicators of significant events during delivery hospitalizations, including: acute myocardial infarction, aneurism, acute renal failure, respiratory distress, thrombotic pulmonary embolism, amniotic fluid embolism, cardiac arrest, eclampsia, heart failure, severe anesthesia complications, sepsis, and ventilation, among others [7]. SMM rates have increased over time, from 0.6% in 1998 to 1.6% in 2011 [5]. Similar to maternal mortality, there are racial/ethnic disparities in SMM. For example, data from seven states revealed higher rates of SMM amongst non-Hispanic Black, Hispanic, Asian/Pacific Islander, and American Indian women, respectively, in comparison to non-Hispanic White women [8].

While infant mortality rates have declined over the past few decades [9], the picture is not much brighter in terms of global comparisons and racial/ethnic, rural–urban, and socioeconomic disparities. Although preterm infant survival rates have increased due to advances in obstetrics and perinatal medicine in the last few decades, the U.S. still has high infant mortality rates (IMR = 5.7; IMRs are calculated as the number of infant deaths per 1000 live births) [10,11]. When comparing the U.S. with the average IMR of 3.9 among Organization for Economic Cooperation and Development (OECD) countries, the U.S. ranks 33 among the 36 OECD countries on this measure [12]. Delving deeper, Black infants have significantly higher mortality rates (IMR = 10.8) than White infants (IMR = 4.6) [10]. In fact, the IMR for Black infants is more than double the rates for Whites and this disparity has persisted for quite some time [13,14]. IMRs are higher for other racial/ethnic minorities as well, including non-Hispanic, Native Hawaiian/Other Pacific Islander (9.4), AI/AN (9.2), Hispanic (4.9), and followed by Asian (3.6) [10]. Socioeconomic differences have also persisted with the mortality rate for infants born to mothers with <12 years of education being significantly higher (135% higher) than those born to women with a college degree [9]. Regarding rural–urban disparities, the highest IMR according to 2013–2015 data is found in rural counties (6.7), followed by small and medium urban counties (6.2), and large urban counties (5.5) [15].

Preterm birth (PTB) and low birthweight (LBW) were among the leading causes of infant mortality within the U.S. in 2018, which exacerbate racial and ethnic disparities in infant mortality [10]. LBW (<2500 g) is a strong determinant for infant mortality, contributing to racial disparities in IMR [16]. In 2019, 8.3% of all births in the U.S. were LBW, with Black infants having the highest rates (14.2%), followed by Asian infants (8.7%) [17]. The U.S. also maintains a high PTB rate (i.e., births < 37 weeks gestation), affecting 10.2% of all births in 2019, which has risen over time, leading to higher rates of death and disability for infants [16,17]. Racial and ethnic disparities for PTB rates exist, with Blacks having the highest rates (14.4), followed by AI/AN (11.6), and Hawaiian/Other Pacific Islander (11.2) [17]. For the purposes of this paper, we will only examine disparities in infant health (≤1 years of age), excluding older children.

## 2. Social Determinants of Health

Social determinants of health (SDoH)—the environmental context and the material and social conditions in which people live, work, and play—help shape proximal risk factors that affect our health, functioning, and quality of life [18,19,20]. SDoH play a key role in creating and sustaining health disparities within the U.S. and occur across multiple levels of influence (i.e., individual, interpersonal, community, and societal) [21]. According to the National Institute on Minority Health and Health Disparities (NIMHD) research framework, SDoH occur across multiple levels of influence, which intersect across multiple domains of influence (biological, behavioral, physical/built environment, sociocultural environment, and health care system) [22] for women and children. The NIMHD research framework levels of influence can help shed light on the multi-faceted and complex nature of maternal and infant health disparities. For example, one study that examined population-level factors and increasing maternal deaths in the U.S. between 1997 and 2012 found that an increased prevalence of obesity and diabetes and other chronic health problems only partially explained the worsening maternal outcomes [23]. This increase in maternal mortality was also attributable to SDoH factors occurring across multiple levels, such as the proportion of women of childbearing age who did not complete high school, the proportion of births among Black women, and the proportion of women who attended fewer than 10 prenatal visits [23]. 

Racism has been defined by the prior President of the American Public Health Association, Dr. Camara Phyllis Jones, as “a system of structuring opportunity and assigning value based on the social interpretation of how one looks (which is what we call ‘race’), that unfairly disadvantages some individuals and communities, unfairly advantages other individuals and communities, and saps the strength of the whole society through the waste of human resources” [24]. Structural racism is a root cause for many SDoH that occur across multiple levels of influence [25,26]. It is racism that is embedded in macrolevel systems where social forces, institutional practices, and ideologies work to reinforce and perpetuate racial and ethnic inequity [27,28,29,30]. Racial residential segregation or redlining, a form of structural racism, has kept people of color from purchasing homes due to unfair lending practices [31]. Although redlining has been illegal since the 1960s, its effects have persisted over time, because it has shaped communities and the resources available. For example, Black women currently living in redlined areas are at greater risk for adverse birth outcomes (e.g., LBW, PTB) [31,32]. This association is further shaped by place-based factors such as access to quality health care and facilities, neighborhood conditions, and environmental exposures (pollution, traffic, heavy metal exposure), which also are associated with poor birth outcomes and maternal morbidity and mortality [31,33,34,35,36,37,38,39].

## 3. SDoH and the Life Course

In order to understand the mechanisms and pathways causing maternal and infant mortality disparities, we must acknowledge that cumulative exposures to SDoH over time, such as racism, segregation, discrimination, insufficient supports provided, and poor quality of health care received, can lead to accelerated biological aging or “weathering” due to the wear and tear of exposures on the body [35,40,41]. For example, research on “weathering” indicates that older maternal age (30–34 or 40+ years) is associated with more rapid increases in low and very low birthweight for racial and ethnic minority women when compared with their White counterparts [40]. Likewise, older Black and Mexican women who give birth experience higher infant death rates than their White counterparts, even after adjusting for sociodemographic and relevant risk factors [41]. SDoH affect the health and well-being of generations across the life course and over time; a mother’s preconception health and social standing are inextricably linked to the health of her future offspring and subsequent generations [28,42,43,44]. The life course theory posits that risk and protective factors occurring during sensitive and critical periods across the life span can have greater impact through their influence on biological systems, thus leading to differing disease trajectories and health outcomes [45,46]. Poor health outcomes typically occur when one is exposed to risk factors or negative events (e.g., stressors such as racial discrimination, heavy metal exposures) without sufficient resources to buffer adverse exposures [47]. Adverse exposures during sensitive or critical periods of development can lead to epigenetic changes, switching on or off genes responsible for chronic disease such as diabetes mellitus or cardiovascular disease [45]. For example, the Barker hypothesis indicates that fetal experiences during critical periods (i.e., fetal programming) in utero and during early infancy can indicate a predisposition for metabolic, endocrine, or cardiovascular diseases, which has implications for not only the developing infant’s life as an adult, but also the mother’s lifelong health trajectory [48,49]. Thus, experiences occurring in a mother’s life prior to pregnancy, stress during pregnancy, and the accumulation of stress across the life course are associated with an increased risk for heart disease and other illnesses for the mother as well as her child as they age [35,50,51,52]. However, protective resources such as positive emotional connections and support, place-based resources (local amenities, access to quality health care, green space), and public policies or programs (safe and adequate housing, adequate nutrition and education, financial support) can help alleviate some of the negative health effects of adverse exposures [49,53,54]. The rest of this paper will critically review the SDoHs leading to disparities in maternal and infant health, drawing on the NIMHD research framework.

## 4. Maternal Health Disparities

The seven leading causal factors implicated in U.S. maternal mortality include thrombotic pulmonary embolism, hemorrhage, hypertensive disorders of pregnancy, infection, cardiomyopathy, cardiovascular conditions, and non-cardiovascular medical conditions [55]. Black women have a higher likelihood of dying from the aforementioned causes than their White counterparts [55,56]. In 2018, a commentary in the *Journal of the American Medical Association* underscored the importance of addressing social inequality as key to reducing high maternal mortality rates [53]. It cited research on specific SDoH, including the link between adverse childhood events and chronic health problems, the cumulative stress of poverty and long-term outcomes, and how racism can lead to weathering or accelerated aging, which is related to increased rates of chronic health problems that may lead to maternal mortality. A review on the relationship between SDoH and pregnancy-related mortality (i.e., deaths which occur within one year of pregnancy) and morbidity indicated a need to evaluate a wider array of determinants—such as the role of socioeconomic and political context (e.g., local policies) or area-level physical and material circumstances influencing maternal outcomes, and the mechanisms that underlie observed associations of determinants [54]. Next, we present a review of the research on various SDOH factors across multiple levels of influence (individual, interpersonal, community, and societal) affecting maternal mortality and morbidity.

### 4.1. Individual Factors

A recent comprehensive review on a variety of individual factors found strong evidence for the impact of race (Black) and ethnicity (Hispanic), lack of health insurance or having public insurance, and lower education on maternal mortality and severe morbidity [54]. The authors of this review noted that there were no studies in their review that evaluated maternal mortality among American Indian women. This comprehensive review also revealed that considerably fewer studies examined the impact of marital status (11 studies), nativity (6 studies), employment status (3 studies), and income (1 study), on maternal mortality and severe morbidity [54]. Two studies that employed national or state analyses on marital status showed unmarried women having higher pregnancy-related mortality rates than married women, and a different study showed that although unmarried White women had higher mortality ratios, there was no significant difference in mortality ratios between unmarried and married Black women [54]. Moreover, one study found that being unmarried slightly increased the odds of severe maternal morbidity. In terms of nativity, one study found that foreign-born Hispanic women had a higher likelihood of pregnancy-related mortality in comparison with U.S.-born Hispanic women, whereas two other studies found no associations [54]. Two studies, in New York City and California, found that foreign-born Hispanic women had higher odds of severe maternal morbidity (e.g., eclampsia and preeclampsia) [54]. The authors of this review did not find convincing evidence linking unemployment or household income with severe maternal morbidity [54]. However, given the paucity of studies on these two variables, no firm conclusions can be drawn. Of the individual factors in this review, health insurance and education are important contributors to maternal mortality and morbidity that could be modified through policy interventions.

Other important individual factors include mental health conditions, such as postpartum depression, history of depressive disorders, anxiety disorders, substance use disorders (SUDs), and other mood or psychotic disorders. In the U.S., postpartum depression (PPD) is a highly prevalent disorder; for example, 13.2% of women experienced PPD in 2018, with higher rates among AI/AN (22.0%), Asian/Pacific Islander (19.2%), and Black women (18.2%) compared with white women (11.4%) [57]. Comorbid SUD and other mental health conditions (depressive and anxiety disorders) are also common in women of reproductive age [58], and there is often a bidirectional relationship with physical chronic conditions and illness [59]. A recent study that examined 2008–2017 Maternal Mortality Committee Review data from 14 states found that among 421 pregnancy-related deaths with determined causes, 11% were due to mental health conditions (with 63% of these due to suicide) [60]. Furthermore, in 67% of these deaths due to mental health conditions, the woman had a history of or current substance use. All the pregnancy-related mental health deaths for which preventability could be determined (*n* = 37) were found to be preventable [60]. These results highlight the importance of mental health screenings for pregnant women and mothers and timely treatment during the perinatal period.

A closely related individual factor is intimate partner violence (IPV), including homicide during pregnancy and the postpartum period. A study using 2018–2019 data on mortality among U.S. females 10–44 years old, pregnant, or within 1 year postpartum revealed 3.62 homicides per 100,000 live births [61]. Moreover, it showed that homicide mortality exceeded by more than twofold all the other leading causes of maternal mortality (e.g., hypertensive disorders, hemorrhage, and infection) during pregnancy and within the first 42 weeks postpartum, with pregnancy-associated homicide being highest among the non-Hispanic Black population and females younger than 25 years [61]. Although the aforementioned study could not evaluate the involvement of IPV in these homicides due to data limitations, it did find that the majority of the homicides took place in the home. In the U.S., more than half of female homicide victims involve IPV, with the vast majority of homicides being perpetrated by a male partner [62]. Impacts of IPV on maternal health are not limited to homicide, but also other negative risk factors such as insufficient or inconsistent prenatal care, poor nutrition, inadequate weight gain, substance use, and increased prevalence of depression [63]. In addition, IPV results in adverse infant health outcomes such as LBW, PTB, and small for gestational age [63]. Routine screening for IPV, engaging patients in prenatal care, and targeted individual interventions (e.g., the Domestic Violence Enhanced Home Visitation Program) have shown promising potential in reducing the adverse impacts of IPV on maternal and infant health [63]. However, further research is needed on the contribution of IPV to racial disparities in maternal mortality and the underlying mechanisms.

### 4.2. Interpersonal Factors

It is important to examine interpersonal factors, including patient–provider interactions, especially given the high preventability of maternal mortality. Most pregnancy-related deaths (65.8%) are preventable, with cardiovascular conditions, hemorrhage, infection, embolism, cardiomyopathy, mental health conditions, and preeclampsia/eclampsia accounting for about 75% of these deaths [64]. Moreover, there is no significant difference in the percentage of preventable deaths between non-Hispanic Black (63.0%) and non-Hispanic White (68.2%) women, nor between Hispanic (61.8%) and non-Hispanic White women [64]. However, racial/ethnic disparities persist; for example, Black and Hispanic women are less likely to receive adequate management of their medical comorbidities and have a higher likelihood of dying from these causes compared with White women with identical conditions [65].

International and national studies generally find that preventable maternal deaths are due to the following provider factors: failure to diagnose, delays in diagnosis, lack of appropriate referrals, and poor documentation and communication [66,67]. However, due to structural racism and low diversity in the U.S. medical workforce [68], these provider factors may disproportionately affect racial/ethnic minority women. Several modifiable provider-level factors have been noted as partial explanations for racial/ethnic disparities in maternal mortality and morbidity, including implicit bias, cultural competence, and patient–provider communication among others [69]. However, we did not find studies in the U.S. that have explicitly examined the contribution of these factors to racial/ethnic disparities in maternal mortality. To elucidate the pathways through which racial/ethnic disparities in maternal mortality and morbidity are generated, more research on the contribution of provider factors should be conducted.

### 4.3. Community Factors

#### 4.3.1. Residential Segregation and Neighborhood Deprivation

Although some studies have examined the relationship of residential segregation with infant mortality and birth outcomes, we did not find any studies in the U.S. that have investigated its relationship with maternal mortality. However, one study in New York City found that pregnancy-related hypertension mediated the relationship between racial residential segregation and low birthweight among Black women [70], and a more recent study in Chicago found a relationship between racial residential segregation and the prevalence of hypertensive disorder of pregnancy among Black women residing in higher poverty neighborhoods [71]. Thus, there is scant but preliminary evidence that residential segregation may be associated with severe maternal morbidity, and perhaps mortality.

In terms of neighborhood deprivation, we found only one study conducted in California that showed an increased risk of pregnancy-associated death in areas of concentrated deprivation (with high proportions of Black and low-income residents), which were measured through calculating census tract-level values of the Index of Concentration at the Extremes [72]. The Index of Concentration at the Extremes quantifies the uneven distribution of privilege by measuring the extent to which an area’s residents are concentrated into groups at the extremes of deprivation and privilege [72]. A few studies from different countries have examined neighborhood deprivation and severe maternal morbidity. A Canadian study found that primiparous women living in neighborhoods of high material deprivation had higher rates of severe maternal morbidity compared with those in low deprivation neighborhoods [73]. A Dutch study reported a 50% increased risk of preeclampsia among women living in deprived neighborhoods [74]. A study conducted in New York City found that women living in zip codes with the highest racial and economic polarization (highest concentration of poor Blacks versus wealthy Whites) experienced the highest severe maternal morbidity rates compared with women living in neighborhoods with the lowest polarization [75]. Thus, there is preliminary evidence showing a potential relationship between neighborhood deprivation and maternal mortality and morbidity.

#### 4.3.2. Rural/Urban Residence

A national study that used data from 2007 to 2015 found that residents of rural areas had a 9% higher probability of severe maternal morbidity and mortality compared with urban residents [76]. One study in Kentucky found interaction effects between rural status and anemia, with rural residence exacerbating the risk for severe maternal morbidity among women with anemia [77]. In general, rural residents experience inadequate access to health services during the perinatal period [78]. A study using national data found that 9% of rural counties lost all hospital obstetric services over the period of 2004–2014, and another 45% of rural counties did not have any hospital obstetric services during this period [79]. Thus, lower access to obstetric and perinatal services may be contributing to rural/urban disparities in maternal mortality and morbidity; however, the causes of these disparities may be multifactorial and need further research. Furthermore, rural communities composed of a higher percentage of Black, Hispanic, and unemployed residents have been shown to experience a higher likelihood of occurrence of hospital closures and loss of hospital obstetric services [80,81]. Thus, examining rural racial and ethnic disparities in maternal morbidity and mortality is also a potential area for future research.

#### 4.3.3. Environmental Factors

We did not find any studies in the U.S. that have explicitly examined the impact of environmental factors on maternal mortality; however, a few have examined the association between some environmental factors and risk factors for severe maternal morbidity. A systematic review and meta-analysis of studies examining the impact of nitrogen oxides (NO_2_, NO_X_), particulate matter (PM_10_, PM_2_._5_), carbon monoxide (CO), ozone (O_3_), proximity to major roads, and traffic density showed that all these air pollutants, except for CO, were associated with an increased risk for pregnancy-induced hypertensive disorders [82]. Another systematic review and meta-analysis of studies examining the association between air pollution exposure and risk of gestational diabetes mellitus found consistent epidemiological evidence for this association [83].

A recent review article of mechanistic studies indicated that chemical exposures via air, water, soil, food, and consumer products (by perturbing biological pathways and processes) and nonchemical stressors (by exacerbating the effects of chemical exposures) may contribute to risk factors for maternal mortality and morbidity [36]. Racial/ethnic minority women experience a higher burden of these chemical and nonchemical exposures [36]. For example, one study showed racial disparities in soil exposure to arsenic (As) and lead (Pb) among pregnant mothers, with non-Hispanic Black mothers having higher exposures to soil concentrations of these substances than White mothers, even after controlling for socioeconomic status and distance to industrial facilities [84]. Thus, a potential area for research is examining whether disproportionate environmental exposures among racial/ethnic minorities may be contributing factors to disparities in maternal mortality and morbidity.

Similar to environmental exposures, we did not find any studies investigating occupational exposures and maternal mortality in the U.S. However, one review of studies conducted between 1966 and 2011 found insufficient evidence on the association of physical work exposures (including working hours, shift work, standing, lifting, and physical workload) with pre-eclampsia and gestational hypertension and called for more research in this area [85]. A more recent review of physical work exposures and pregnancy outcomes found an association between lifting objects ≥11 kg and preeclampsia [86]. Another review that focused on occupational shift work found that working rotating shifts was associated with increased odds of preeclampsia and gestational hypertension compared with working fixed day shifts [87]. One study from New Jersey showed that intensive physical activities at work during pregnancy were associated with an increased risk of gestational diabetes [88]. However, there is profound occupational segregation in the U.S. labor force [28]. The distribution of occupations among U.S. adults is strongly patterned by social identities consistent with populations that experience health disparities (e.g., gender, race/ethnicity, socioeconomic status, and rural/urban residence). For example, African Americans and Hispanics are the least likely to be in managerial and professional jobs and most likely to be in service and blue collar jobs [89]. A national study found consistent associations between working in race-segregated occupations and poor worker health [90]. There is also segregation within the workplace by social identity, which can result in large differences in exposure to workplace hazards [28]. However, we did not find studies that specifically examined whether differences in maternal occupational exposures within or across workplaces explained disparities in maternal mortality and morbidity. More studies are needed on the maternal exposome—which measures all the exposures of an individual in a lifetime, including those from environmental and occupational sources, and how those exposures relate to health—in order to understand the mechanisms and pathways through which environmental and occupational exposures impact certain risk factors for maternal mortality and morbidity such as diabetes, cardiovascular events, obesity, and other diseases [36]. In addition, future research should examine whether differences in environmental and occupational exposures explain disparities in maternal health.

### 4.4. Societal Factors

Structural racism is a root cause for many SDoH [25], including some of the aforementioned individual, interpersonal, and community factors, as well as societal factors. For example, in the previous section on community factors, we present preliminary evidence of the contribution of residential segregation and neighborhood deprivation, both of which are caused by structural racism, to racial disparities in maternal mortality and morbidity. Structural racism refers to the cumulative ways in which societies promote racial discrimination through systems that mutually reinforce each other such as housing, education, media, employment, health care, etc. [91]. Thus, many racial and ethnic minorities in the U.S. face an inherited unequal distribution of resources and enduring discrimination that limits their access to quality education and jobs, political power, healthy neighborhoods, and quality health care [92]. In this section, we highlight the literature on the following societal factors in relation to maternal mortality and morbidity: hospital care, health insurance expansions and access to care, and access to paid maternity leave.

#### 4.4.1. Hospital Care

A consistent finding across studies performed in different states is that women who gave birth in majority Black-serving hospitals experienced higher severe maternal morbidity rates than those in low-Black-serving hospitals [93,94,95,96,97]. However, there were mixed findings regarding Hispanic-serving hospitals [93,97]; one study found higher maternal in-hospital mortality rates for majority Black-serving hospitals only among non-teaching hospitals [98]. In New York City, Black women had a higher likelihood of giving birth in a hospital with higher risk-adjusted severe maternal morbidity rates than White women [96]. The excess risk among Black women was not fully accounted for, even after taking into consideration hospital teaching status, having a level 3/4 nursery, private ownership, and a very high delivery volume [96]. In another study, the hospital percentage of Medicaid deliveries was associated with higher rates of severe maternal morbidity [95]. Giving birth in rural hospitals was associated with a higher risk of readmission [99] and longer hospitalization stay [100] than delivering in urban hospitals. Thus, hospital characteristics are potential contributing factors to maternal mortality and morbidity disparities.

Research has not explicitly examined differences in access to quality care as a determinant of racial disparities in maternal mortality and morbidity. However, a review by Howell and Zeitlin provides an overview of the evidence relating hospital quality with maternal mortality and morbidity and discusses the potential pathways through which these associations may result in disparities, including between- and within-hospital differences [69]. Thus, this is a potential area for future research that may uncover further SDoH leading to disparities in maternal mortality and morbidity.

A recent review called for a unified approach to address the contribution of quality of hospital care to racial/ethnic disparities in maternal and infant health outcomes [65]. Both mothers and infants from minority populations may experience worse perinatal outcomes because of deliveries occurring in poorer quality hospitals (between-hospital differences), receiving inferior quality of care than non-Hispanic Whites in the same hospital (within-hospital differences), or have health and social risks that hospital care cannot address [65]. More work is needed to evaluate clinical protocols through the lens of the mother–infant dyad, integrate findings into obstetrics and neonatal care; develop quality metrics for the mother–infant dyad; and understand how disease processes affect the mother–infant dyad across the life course [65].

Currently, initiatives to address quality of care for mothers and infants are separate and not well-evaluated. For example, the Alliance for Innovation in Maternal Health has stimulated the implementation of quality improvement (QI) initiatives using maternal safety bundles in 18 states and more than 800 hospitals to address disparities in maternal mortality [53]. The main outcome of implementing these QI initiatives that has been evaluated so far has been the timely treatment of severe hypertension, which has increased from 42% to 79% [101]. On the other hand, continuous quality improvement (CQI) initiatives in the NICU have experienced challenges in implementation and sustainability and have indicated mixed results in terms of improving NICU outcomes [102]. Thus, more research is needed to evaluate quality improvement initiatives and their impact on maternal and infant health disparities, ideally through a mother–infant dyad lens.

#### 4.4.2. Insurance Coverage Expansions

Medicaid is the largest payer for maternity care in the U.S., providing coverage for pregnant and postpartum women living at 138% of the federal poverty line (FPL), although only provides coverage for mothers up to 60 days postpartum despite a considerable number of maternal deaths and complications occurring after this time [103,104]. Under the Affordable Care Act coverage expansions in 2014, states were given the option to expand Medicaid eligibility to all adults under the age of 65 up to 138% of the FPL, regardless of pregnancy status [105]. Being uninsured before pregnancy has been associated with a higher prevalence of preconception health risk factors (e.g., cigarette smoking, lower physical activity, higher BMI, unwanted pregnancy, lower pre-pregnancy multivitamin use) [106]. By 2019, the 17 states that opted not to expand Medicaid had a disproportionate share of uninsured women of childbearing age (53.8% of the 7.5 million uninsured women) [107]. In addition, Hispanic women living in non-expansion states in 2019 had the highest uninsured rates amongst all racial/ethnic groups [107]. A recent study that evaluated the impact of Medicaid expansion under the ACA found that it was associated with a lower total MMR by 7.01 maternal deaths per 100,000 live births relative to non-expansion states [108]. Moreover, when stratifying by race/ethnicity, non-Hispanic Black mothers had the highest decreases in total MMR (by 16.27), followed by Hispanic mothers (by 6.01), whereas the decreases for White mothers were not significant. Thus, state Medicaid expansions could be contributing to reductions in maternal mortality disparities [108]. Further research should examine the mechanisms through which Medicaid expansions contribute to decreases in maternal mortality rates and corresponding decreases in racial/ethnic disparities.

#### 4.4.3. Paid Maternity Leave Policies

In the U.S., two-thirds of first-time mothers are employed, with most working full time through the final months of their pregnancies [109]. However, one-quarter of mothers are back to work two weeks after childbirth [110]. Notably, the U.S. is one of only two countries in the world (the other is Papua New Guinea) that do not have a national paid leave law [111]. Thus, women’s access to paid leave is contingent on their employer’s policy and/or the state in which they reside. No studies to date have examined the impact of paid maternity leave on maternal mortality and severe maternal morbidity. However, mothers need time to recuperate and adjust after birth, and research has consistently shown that access to paid leave is associated with better maternal mental health [112,113]. Updated guidance by the American College of Obstetricians and Gynecologists (ACOG) pointed out that the immediate period after childbirth, or “the fourth trimester” (3 months postpartum), is key for postpartum care because many pregnancy-related deaths and complications arise during this time [114].

In addition, leave policy reform in Norway, which provided four months of paid maternity leave, was associated with a range of better outcomes for Norwegian mothers, including lower body mass index, lower obesity, lower blood pressure and pain, improved mental health, and better self-reported health [115]. The lack of a national paid leave law in the U.S. lends itself to racial and ethnic disparities in access to leave benefits. For example, Black and Hispanic mothers are more likely than their White counterparts to report being fired by an employer for taking leave after childbirth or quitting their jobs after childbirth [116]. Three out of every ten discrimination claims were filed by Black women between 2011 and 2015, including being fired for taking maternity leave, being denied a promotion or raise due to pregnancy, having inadequate maternity leave allowance, and having to endure physically taxing work conditions or extreme manual labor during pregnancy [117]. However, in the presence of a paid leave policy, there is evidence that these racial disparities decrease. For example, prior to California’s paid leave law, Black women took an average of one week of leave after childbirth compared with four weeks for White women, whereas after this law’s implementation, the average maternity leave taken by both Black and White women increased to seven weeks [118]. Thus, uneven access to paid leave policies in minority populations and the disparities in maternal mortality and morbidity is a potential area for future research.

## 5. Infant Health Disparities

Racial and ethnic disparities in U.S. infant health occur before, during, and shortly after birth. The leading causes of infant mortality within the U.S. in 2018 were congenital malformations, disorders related to short gestation, i.e., PTB, and LBW, and maternal complications [10]. LBW and PTB infants are more likely to experience neonatal intensive care unit (NICU) stays, which account for one-half of infant hospitalizations costs and one-quarter of pediatric care costs in the U.S. [119]. Furthermore, racial and ethnic minority infants, especially Blacks, experience disparities in NICU care [120,121]. Disparities experienced during the early years of a child’s development increase the risk for poor health and educational outcomes across the lifespan [122]. Next, we present a review of the research on various SDOH factors across multiple levels of influence (individual, interpersonal, community, and societal) affecting infant mortality and morbidity.

### 5.1. Individual Factors

Most infant health individual factors overlap with maternal factors, due to the current risks associated with birthing in the U.S. such as inadequate access to health care, exposure to racism and discrimination, and lower maternal educational attainment, which contribute to disparities in infant mortality and adverse birth outcomes [35,123,124,125]. However, in international samples, women with fewer years of education have an increased likelihood of experiencing preterm birth, suboptimal fetal growth, still birth, and infant mortality [126]; in the U.S., infant mortality rates among college-educated Black parents are twice those of White parents [127]. In fact, this complex interplay between socioeconomic status and racial disparities is demonstrated in a study that found significant interactions between race and income, maternal education, and paternal occupation and racial disparities in the risk of preterm birth [128]. This study showed a decrease in the difference in preterm delivery rates between Black and White women as community poverty and social deprivation increased; moreover, differences were no longer detected when census tract-level poverty was above 25% [128]. In addition, some studies show that having a foreign-born mother may provide some protection against poor birth outcomes [124]. Thus, studies examining maternal individual factors and infant health outcomes within a U.S. context will need to use an intersectionality lens in order to detect the complex interactions of maternal individual factors leading to infant racial/ethnic health disparities.

### 5.2. Interpersonal Factors

Structural racism and implicit bias pervade medical care; racial and ethnic minority patients report receiving worse care than their White counterparts [68]. Additionally, there are fewer medical providers from diverse backgrounds even though higher levels of trust and satisfaction are reported from patients who receive care from a physician of the same race [68,129]. Culturally competent neonatal and perinatal medicine training during graduate medical education can help medical trainees provide appropriate medical management for neonates, identify disparities in neonatal care, and help develop quality improvement initiatives to reduce infant care disparities [130].

Discrepancies in infant care exist early on, which cannot be ameliorated by hospital care [69]. Where infants are born can affect the quality and type of care they receive. Delivering in hospitals with a poorer quality of care can influence infant health [35]. Considerable racial and ethnic disparities exist within and between quality NICU care, especially for Black infants, often due to modifiable quality measures such as timely eye examinations and the consumption of human milk at discharge [131,132]. Likewise, families with children in the NICU reported experiencing suboptimal care that was neglectful and judgmental from NICU staff based on characteristics such as race, language, ethnicity, or culture [133]. These discrepancies could be targeted by providing more culturally competent care and ensuring racial concordance between the newborn physician and the infant, especially for Black infants to reduce their risk for infant mortality [131,134].

### 5.3. Community Factors

#### 5.3.1. Residential Segregation and Neighborhood Deprivation

Several studies in the U.S. examined the relationship between racial residential segregation and infant mortality and found adverse effects on Black infant mortality [135,136,137,138]. In addition, a meta-analysis of studies that examined racial residential segregation and adverse birth outcomes found that segregation was associated with an increased risk of PTB and LBW among Black mothers, but had little or no association with birth outcomes among White mothers [139]. More studies are needed to examine the relationship between segregation and infant mortality, using more recent data from the U.S., and to identify the specific mechanisms through which residential segregation impacts disparities in infant health outcomes.

In terms of neighborhood deprivation, one study in California showed an increased risk of infant mortality and PTB in areas of concentrated deprivation (with high proportions of Black and low-income residents), which were measured through the Index of Concentration at the Extremes (ICE) [72]. Another study in New York City found, using ICE, that women residing in areas with the least privilege were more likely to experience a PTB or infant mortality as compared with women residing in the most privileged areas [140]. Other neighborhood-level based SDoH factors, such as neighborhood poverty level, structural deterioration, areas impacted by mass incarceration, and historical redlining, are associated with adverse birth outcomes (i.e., LBW and PTB) especially among Black infants and mothers [31,141,142,143]. More studies are needed to confirm the relationship of neighborhood deprivation with infant mortality and PTB, to examine its relationship with LBW, and to investigate the mechanisms through which neighborhood deprivation affects infant health outcomes and contributes to racial/ethnic and socioeconomic disparities.

#### 5.3.2. Rural/Urban Residence

IMR varies by location: states in the South (e.g., Alabama, Arkansas, Florida) and Midwest (e.g., Illinois, Indiana, Michigan) had IMRs that were significantly higher than those in the Northeast and the Northwest of the U.S. [10]. To understand what drives and sustains these disparities, we must consider SDoH place-based factors related to IMR. Research indicates that IMRs are higher in rural areas due to socioeconomic disadvantage, higher levels of risk factors, and limited access to health care [144].

Overall, IMRs are higher for Black infants regardless of residence (urban or rural) [145,146]. However, when adjusting for county-level structural racism indicators (e.g., income inequality, jail incarceration rates, educational attainment disparities), striking racial disparities emerge in urban areas [146]. Specifically, residing in an urban county was associated with a 7–9% increase in Black IMRs and a 6% decrease in White IMRs. Thus, the complex interplay between race and rural/urban residence should be taken into consideration, and more research is needed to clarify the relevant mechanisms and risk factors for infant mortality and morbidity.

#### 5.3.3. Environmental Factors

Multiple studies in the U.S. have shown associations of prenatal exposures to air pollution with increased risks of PTB and LBW [147,148]. Moreover, some studies showed racial/ethnic inequalities in these airborne exposures, with Blacks and Hispanics suffering higher exposure burdens than Whites [149]; one study in Florida showed that exposure to air particulate pollutants was associated with increased odds of PTB, LBW and very low birthweight, with Black women having 68–300% higher increased odds for all morbidity outcomes compared with White women [150]. One study in California found evidence of an association between long-term exposure to fine particulate matter air pollution and postneonatal infant mortality [151]. In addition, epidemiological studies show that prenatal lead exposure is associated with higher risks of PTB and LBW [152,153,154]. Despite the general decline in lead levels throughout the U.S. between 1999 and 2012, average levels have been persistently higher among Black compared with White adults and children [155]. One study that examined the relationship between a measure of cumulative environmental exposure (across air, water, land, sociodemographic, and land domains), an Environmental Quality Index, for U.S. counties from 2000 to 2005, found that poorer environmental quality was associated with increased odds of infant mortality among Black and Hispanic mothers, and rural status was associated with increased odds of infant mortality among Hispanic mothers [156]. Thus, although there are some U.S. studies showing that environmental exposures are associated with poor infant health outcomes and showing disparities by race/ethnicity and rural status in these exposures, very few studies have examined whether disparities in exposures or in vulnerability explain disparities in infant health outcomes.

We did not find studies in the U.S. that examined the association between occupational exposures and infant mortality. However, in general, studies have shown that pregnant women who work in physically demanding jobs, such as those that require them to stand for long hours, lift heavy objects, perform shift work, or work nights, have a higher likelihood of having PTB and LBW infants [157,158,159]. A study conducted in the Netherlands found that maternal occupational exposure to polycyclic aromatic hydrocarbons, phthalates, alkylphenolic compounds, and pesticides is associated with impaired fetal growth and decreased placental weight [160]. Another study in Sweden found that women with low levels of job control had higher risks for low birthweight, very low birthweight, small for gestational age, all preterm, very preterm, and extremely preterm births, and those exposed to job hazards had higher risks for very low birthweight and extremely preterm birth [161]. One study in the U.S. found that being underemployed or involuntarily working part-time was associated with delivering LBW infants [162]. However, we did not find studies that specifically examined whether differences in maternal occupational exposures within workplaces or across occupations explained disparities in infant health outcomes. Future research should examine the relationship between maternal occupational exposures and infant mortality and morbidity and whether differences in these exposures explained disparities in infant outcomes in the U.S.

### 5.4. Societal Factors

Societal upstream factors such as housing and employment policies collectively contribute to inequities in birth and infant health outcomes [163]. Policies guiding mortgage lending practices and community development shape communities, and more specifically, the resources, exposures, and social capital they contain. For instance, safe, stable, affordable, and quality housing has a direct relationship with physical and mental health, and is a key SDoH in health promotion [164]. Racial and ethnic minority families are more likely to live in areas with concentrated poverty, experience housing insecurity and housing segregation which contribute to greater disparities in infant mortality and birth outcomes [163,164,165]. One study comparing state-level measures of structural racism and Black infant mortality found that for all states, increasing racial inequity in unemployment was associated with a 5% increase in Black infant mortality [14]. Furthermore, high levels of racial housing segregation are associated with greater disparities in Black/White IMRs, specifically in urban areas [163]. Racial residential isolation (a measure of residential segregation) was strongly associated with racial inequity in IMRs, with the Black IMR being 20% higher than the White IMR [163].

Health care access is a key factor contributing to disparities in infant mortality and birth outcomes. In the U.S., employment status plays an important role in mother’s health insurance access and parental leave benefits, if any, because most individuals access these resources through their employer. Thus, it is no surprise that maternal unemployment is associated with adverse birth outcomes in the U.S., because this limits access to resources such as insurance, quality medical care, and healthy foods, and can increase stress [166].

Longer duration of paid maternity leave, which enables workers to take time away from work to heal from childbirth, take care of the newborn and breastfeed, and adhere to postpartum care and child wellness visits, has been associated with decreases in perinatal, neonatal, infant, and child mortality in member [167] and non-member [168] countries of the OECD, increased breastfeeding [169], and improved maternal mental health [112]. Based on this evidence, in 2018, the ACOG endorsed at least six weeks of fully paid leave for all new mothers [114]. In 2019, the President signed the Federal Employee Paid Leave Act, which provides 2.1 million federal workers with up to 12 weeks paid leave following childbirth, adoption, or fostering. Evaluation of the impact of new paid leave initiatives in the U.S. on disparities in infant health outcomes and the mechanisms through which these initiatives affect outcomes is needed.

Social safety-net programs, such as Medicaid, Children’s Health Insurance Program (CHIP), and the U.S. Department of Agriculture’s Special Supplemental Nutrition Program for Women, Infants, and Children (WIC) program, have been shown to improve perinatal and birth outcomes as well as breastfeeding rates [170,171,172,173]. Medicaid and CHIP, joint state- and federal-based programs, provide free medical coverage for low-income pregnant mothers and children. WIC is a federally funded grant program given to states to protect the health of women, infants, and children (0–5 years) living in low-income households and currently serves about half of all U.S.-born infants [174]. WIC participants receive nutritional assistance, lactation support, immunization screening, connections to Medicaid, and health care referrals [174]. Participation in WIC is associated with improved health (e.g., improved birth outcomes, reduced rate of hospitalizations and NICU stays) among Black and Hispanic infants and reduced racial and ethnic disparities in infant health [175]. Racial and ethnic minority women are disproportionately enrolled in Medicaid during pregnancy, and roughly 50% of WIC children are Hispanic or Latino and 20% are Black [176,177]. Medicaid coverage with WIC use is associated with reduced PTB risk and infant mortality [171]. Under the Affordable Care Act, some states have expanded Medicaid coverage to 12 months postpartum. States that expanded Medicaid indicated a lower uninsured rate among mothers who gave birth as well as reducing racial, but not ethnic disparities in PTB and LBW [178]. More studies are needed to evaluate the impact of eligibility expansions for WIC and Medicaid expansions on disparities in infant mortality and morbidity.

## 6. Maternal and Infant Health Disparities: Research Gaps

We reviewed the literature on social determinants of maternal and infant health and identified several areas where more research could be conducted. Several individual maternal factors could be further explored, such as sexual and gender identity status, nativity, income, employment status, marital status, mental health, and experiences of IPV, and the interactions of these factors with race/ethnicity. In terms of interpersonal factors, implicit bias from providers, patient–provider communication, racial concordance between the provider and patient, and strategies to improve cultural competence should be examined further in relation to their impact on disparities in maternal and infant mortality. In terms of community factors, there is a dearth of research on the impact of environmental and occupational exposures on maternal and infant mortality and morbidity; similarly, there is only preliminary evidence regarding the effects of residential segregation and neighborhood deprivation, and scarce research on the mechanisms through which they impact disparities. Additionally, more research is needed on how differential access to obstetric and perinatal care leads to rural and urban disparities in maternal and infant health and on the interactions between race/ethnicity and rural/urban residential status. Societal factors that could be considered for future research include the impact of structural racism through limited access to quality education and jobs, access to quality care, and expanding social policies and programs such as paid maternity leave, housing, Medicaid, and WIC. In addition, research could examine the impact of combined approaches to address infant and maternal mortality disparities through continuous quality improvement initiatives.

It is imperative to understand the mechanisms and pathways through which SDoH create health disadvantage across the life course, ultimately affecting maternal and infant mortality [44]. In our review, we found that Black mothers and infants fare the worst in terms of health outcomes, with preliminary research pointing to upstream causes such as the accumulation of SDoH experienced due to structural racism across the life course. However, there are other minority populations, such as AI/ANs, whose disadvantage is not easily detected in studies due to small sample sizes. Thus, there is a need to conduct research that specifically targets these populations and identifies the mechanisms through which disparities in infant and maternal mortality are created and sustained. In addition, maternal and infant health disparities research should examine the interactive effects of SDoH across multiple levels (individual, interpersonal, community, and societal) and across different life stages, and identify protective factors and resources that may buffer against adverse effects. Such research will require devising new composite measures that capture these complex interactions and cumulative exposures across the life course, while utilizing the appropriate study designs and analytical methods.

Recent events such as the SARS-CoV-2 (COVID-19) pandemic have put further stress on families and children, increasing mental health issues and highlighting systemic inequalities, leading to increased disease burden and mortality among racial and ethnic minorities in the U.S. [47]. For example, preliminary data from the U.S. and worldwide, collected by the INTERCOVID Multinational Cohort Study, show higher rates of severe maternal morbidity, maternal mortality, preterm birth, and severe neonatal morbidity during the pandemic, especially among mothers with COVID-19 infection [179]. In addition, the maternal mortality rate in the U.S. spiked during the first year of the pandemic (23.8 in 2020 vs. 20.1 in 2019), with both non-Hispanic Black and Hispanic women experiencing significant increases in maternal mortality compared with the previous year and non-Hispanic Black women having 2.9 times the mortality rate of non-Hispanic White women [180]. These data reiterate the importance of addressing the systemic inequalities underlying the disparities in maternal and infant mortality and morbidity. COVID-19 and recent events have also highlighted the fact that climate change is an important SDoH to consider for maternal and infant health [181]. As climate change worsens, we will experience longer and hotter summers, more frequent and intense storms, rising sea levels, and poorer air quality, which will lead to adverse health outcomes, increased health care costs, and will ultimately disproportionately impact women and children living in poverty [181,182]. Such changes stress the importance of conducting research on upstream SDoH to not only ameliorate disparities in maternal and infant health, but also buffer against the potential impacts of climate change.

## 7. Conclusions

We found that Black mothers and infants fare the worst in terms of health outcomes, likely due to SDoH experienced as a result of structural racism across the life course. This critical review indicates that upstream SDoH are important contributors to disparities in maternal and infant mortality. More research is needed on the effectiveness of policies and programs such as CQI initiatives for the maternal–infant dyad, paid maternity leave, quality, stable, and affordable housing, and the expansion of social safety-net programs. Finally, it is important to address research gaps in individual, interpersonal, community, and societal factors, because they affect maternal and infant mortality and related disparities.
